# Emotionality differences between a native and foreign language: theoretical implications

**DOI:** 10.3389/fpsyg.2014.01055

**Published:** 2014-09-23

**Authors:** Catherine L. Caldwell-Harris

**Affiliations:** Psycholinguistics Laboratory, Department of Psychological and Brain Sciences, Boston UniversityBoston, MA, USA

**Keywords:** bilingualism, emotion, context dependence, embodiment, language acquisition

The topic editors, Cornelia Herbert and colleagues, have noted that language has historically been assumed to be independent from emotions. The historical backdrop to this is the long reign of faculty psychology, which viewed the human mind as composed of discrete abilities (see discussion in Barrett, 2013). The mental modularity popularized by Chomsky (1965) and Fodor (1983) continued this view following the cognitive revolution of mid-century. Emotion had no role in information processing psychology, leading to its neglect in the cognitive sciences (Cromwell and Panksepp, 2011). Indeed, the classic emotion-cognition divide has been criticized in the past decades by theorists who are otherwise not natural allies (e.g., Damasio, 1994; Cromwell and Panksepp, 2011; Lindquist, 2013). An alternative to faculty psychology is psychological construction (Lindquist, 2013). On this view, mental abilities and mental states like emotions are constructed from the dynamic interaction of physiological states, situation-specific information, and conceptual knowledge.

In the modular view of mind, emotion and language should have little overlap in their processes and representations. However, according to psychological constructivism, an emotional reaction can be influenced by any aspect of the on-going situation, such as the language being spoken, which is the topic of this commentary.

I describe here findings on the emotionality differences between a native and a foreign language. Bilingual speakers ^1^ frequently report that swearing, praying, lying, and saying *I love you* feel differently when using a native rather than a foreign language (see, e.g., Pavlenko, 2005; Dewaele, 2010). My goal is to highlight the relevance of this body of work for the theoretical assumptions regarding language-emotion independence.

## When and why is a first language more emotional?

An emotionality advantage for native languages has been documented using diverse techniques, as recently discussed in a comprehensive review paper (Pavlenko, 2012). For example, in a European study using a variety of L1-L2 pairings, advertising slogans were judged to be more emotional when the messages were written in the native language rather than respondents' L2 (Puntoni et al., 2009). Anooshian and Hertel (1994) found emotion-memory effects for L1 but not L2 words, among Spanish-English bilinguals.

Reduced emotionality in the L2 has also been found in studies that use emotion words to interfere with processing. Colbeck and Bowers (2012) compared emotion word processing in native Chinese speakers and native English speakers using an English attentional-blink task. The native English speakers showed a strong blink following a taboo distractor, while Chinese speakers of English as a second language showed a blink that was reduced in size, consistent with being able to more easily ignore the taboo distractor. Other examples of improved performance because of reduced L2 emotionality have been found using decision making tasks. In two studies by different research teams, bilingual speakers made slightly more rational decisions when evaluating vignettes written in a foreign language (Keysar et al., 2012; Costa et al., 2014; see also findings about moral dilemmas, Costa et al., 2014).

Laboratory studies measuring skin conductance amplitudes have corroborated these findings (Harris et al., 2003). An important qualification was obtained by studying early, sequential bilinguals, who learned Spanish first from their parents and English second from peers and schooling in American society (Harris et al., 2006). For these bilinguals, their first language was not their most proficient language. They had similar electrodermal responses for emotional phrases in their two languages. One implication (which needs additional empirical support) is that both early age of acquisition and high proficiency are required to show an emotionality advantage. That is, if only age of acquisition were sufficient to show heightened electodermal responses, then the heritage language learners should have shown stronger emotions to Spanish phrases. If only proficiency mattered, then this group should have shown stronger emotionality responses to L2-English. A comparison group of bilinguals for whom L1-Spanish was both the first learned and most proficient language revealed higher skin conductance responses for childhood reprimands in L1 than in L2. This suggests that L1/L2 emotionality differences are strongest when L1 is the native language and L2 is a less proficient, foreign language.

In addition to early age of acquisition and high proficiency, emotional resonances are stronger when language is learned via immersion, rather than from classroom learning (Dewaele, 2010). Another important factor is high usage frequency (Degner et al., 2011). In the broader literature on L1/L2 effects, these four factors are linked in reciprocal, causal relationships, and indeed, are important for determining individual differences in bilingual experiences and abilities. Early age of acquisition typically results in high proficiency; high proficiency usually leads to frequent use. Frequency of use improves proficiency; immersive learning leads to higher frequency of use and better proficiency.

Note that there have been inconsistences in laboratory tasks of L1/L2 emotionality differences. Several studies have failed to replicate Anooshian and Hertel's emotion-memory effects, with Ferré et al. (2010) reporting no recall effects as a function of L1/L2 status (see also Ayçiçegi-Dinn and Caldwell-Harris, 2009). Similar interference was found for L1 and L2 on an emotional Stroop task (Eilola et al., 2007). When Eilola and Havelka (2011) recorded skin conductance during a taboo Stroop task, they found similar interference effects of the taboo words in L1/L2, but L1 taboo words nevertheless elicited larger autonomic responses than did L2 taboo words.

## Causes: why are emotional resonances strongest when a language is acquired early and learned to high proficiency?

Intuitively, it makes sense that a language learned in childhood will carry strong emotional resonances. The family context of learning means that everyday language carries the full range of human emotions. A mechanism for connecting the physical experience of emotion with specific phrases and words is amygdala-mediated learning. Early language develops at the same time as emotional regulation systems (Bloom and Beckwith, 1989). It is thus plausible that utterances that are learned early become tightly connected with the brain's emotional system. However, second languages can also come to feel emotional, if they are used frequently and are learned via immersion rather than in the classroom (Dewaele, 2010; Degner et al., 2011). This is why I proposed that the primary causal factor is the context in which a language is learned and used (Harris et al., 2006). Words and phrases come to have a distinctive emotional feel by virtue of being learned, or habitually used, in a specific emotional context.

My theoretical proposal is that using a language in emotional contexts provides it with emotional resonances because human experiences are learned and stored in a context-dependent manner. This view is consistent with episodic trace theories of memory (Hintzman, 1986), encoding specificity (Tulving and Thompson, 1973), language-specific autobiographic recall of memory (Marian and Kaushanskaya, 2004, 2008), and psychological constructivism broadly construed (Lindquist, 2013). With context-dependent learning, distributional analysis sorts out, via exposure to many examples, which aspects of the overall meaning most frequently co-occur with specific words and phrases (e.g., McClelland et al., 1986). An alternative view is that frequency of use is what matters rather than contexts of use (e.g., Puntoni et al., 2009; Degner et al., 2011). I suspect the frequency view and the contexts of learning view are actually highly similar perspectives and make different predictions only in rare cases. My reasoning is that frequent usage entails emotional usage. Human social lives, which are mediated by communication, are highly emotional. If there are situations of frequent use of an L2 in low-emotion environment, then my theory predicts that these L2 users will experience their L2 as low in emotional resonances.

One of the strengths of the “emotional contexts of learning” hypothesis is that it accommodates the idiosyncratic learning histories of individual speakers. In a group study on emotional word processing, a word such as *snake* will elicit different emotional reactions depending on individuals' personalities, experiences with snakes and cultural backgrounds. An implication is that we can have two (complementary) ways of studying L1/L2 emotionality effects. We can take average responses across a group of bilingual speakers, by examining language that most people find emotional, such as parents scolding children (childhood reprimands), peers insulting each other (insults), or people expressing love, praise, appreciation (endearments). When my colleagues and I used these categories of emotional expressions, we thus studied common situations where these phrases are learned and used (Harris et al., 2006). But in these studies, individual experiences that deviate from group trends are ignored and treated as noise.

A second method is to interview people about their idiosyncratic experiences. What specific phrases did your parents say to you? How did authority figures speak to you as a young adult? What did a romantic partner tell you that you appreciated? The prediction is that emotionality will be greater for the language that was used and/or learned in these situations. Although this interview technique has not yet been used, immigration narratives revealed that emotional language varies with individual experiences (Marian and Kaushanskaya, 2008).

## Theoretical implication: vocabulary and grammar are not context-independent

To move beyond behaviorists' focus on imitation as the main route to learning, Chomsky (1965) and theorists of the mid-20th century emphasized that linguistic expressions are primarily a result of applying abstract rules. They characterized language as a parsimonious symbol system, a type of mental algebra. The language learner had to strip away words' context to construct context-independent vocabulary. Learners must ignore extra-linguistic aspects of sentences in order to construct an abstract grammar.

The Chomskyan theoretical view dove-tailed with the intuition that many people have, which is that words are containers for meanings. Reddy (1979) has labeled this the conduit metaphor, referring to the belief that language, phrases and sentences are the containers for speakers' meanings and thoughts. These containers are then sent to conversation partners, who extract and thus possess the meaning. Examples provided by Reddy include the common request to “put your feelings into words.”

An inference from the conduit metaphor is that, as long as two phrases are translation equivalents, they should deliver the same meaning. However, “same meaning” is itself open to interpretation. Consider the case where an English native speaker has learned French in a classroom context. When hearing *Je t'aime*, the phrase doesn't deliver the same emotional punch as *I love you* (as documented by Dewaele's study of multilingual speakers' report of I love you expressions; see also Caldwell-Harris et al., 2013). If “extracting and possessing the meaning” includes the totality of mental states that form as a reaction to hearing a phrase, then the I love you examples (and other emotionality effects) falsify the conduit metaphor. However, the Chomskyan tradition has generally advocated a narrow view of meaning, confining it to the sense of words, not their richer connotations. If the meaning of words is confined to what is involved in identifying translation equivalents, then the conduit metaphor can be preserved.

One reason to retain the conduit metaphor (and the narrow definition of meaning) is if the conduit metaphor is the only way we have of understanding how symbols convey information. But other conceptions are present in the research literature and in everyday use. Reddy's (1979) description of how language actually works to provide meaning is called the toolmaker paradigm. Words and phrases are not containers of meaning, but clues that hearers' use to infer speakers' communicative intent. On this view, Je t'aime doesn't deliver the same emotional punch to the classroom French learner as *I love you*, because the phrase isn't a container for the feeling expressed by *I love you*. It's a tool speakers use to guide hearers to an interpretation. In the case of foreign language learners, L2 phrases are imperfect tools for activating the meanings that would automatically be elicited by the same phrase in a native language.

An advantage of discussing the relevance of emotionality differences to the conduit metaphor is that the conduit metaphor and objections to it are a bit abstract. L1-L2 emotionality differences lend concreteness to Reddy's classic critique of the container metaphor.

These arguments in turn have their theoretical implications, including how context is represented. A second theoretical implication of L1/L2 emotionality effects is that words and phrases gain meaning from sensorimotor and emotional embodiment. Both of these are discussed further in Caldwell-Harris (2014).

## Conclusions

Beyond the theoretical implications, understanding L1/L2 emotionality effects is important for bilinguals who may wonder why these effects exist, or may wonder why these effect don't exist for them. Emotionality effects are relevant for monolingual speakers whenever they interact with bilinguals who are using the language that for them is later-learned or less-proficient. And finally, they are important because they challenge us to confirm, refute, or extend our theories about the relationship between language and emotion.

### Conflict of interest statement

The author declares that the research was conducted in the absence of any commercial or financial relationships that could be construed as a potential conflict of interest.

## Footnotes

^1^To be as inclusive as possible, I follow the common practice of identifying bilingualism as either having good proficiency in more than one language, or of regularly using more regularly using more than one language, regardless of proficiency. The first language (L1) is defined to be the chronologically first acquired language, with “second language” meaning a language acquired after the L1 (see Dewaele, 2010). A foreign language is a language acquired primarily via classroom learning, and not a language spoken in the learners' community.
