# Erratum on: Emotionality differences between a native and foreign language: theoretical implications

**DOI:** 10.3389/fpsyg.2015.00437

**Published:** 2015-04-02

**Authors:** Frontiers Production Office

**Affiliations:** Frontiers Production Office, FrontiersSwitzerland

**Keywords:** bilingualism, emotion, context dependence, embodiment, language acquisition

Reason for Erratum: Recognition of an additional reviewer.

Due to an oversight, the following reviewer Lowri Mair Hadden (Bangor University, UK), who jointly reviewed the paper with her supervisor Debbie L. Mills (Bangor University, UK), was not acknowledged on the publication. This error does not change the scientific conclusions of the article in any way. The publisher apologizes for this mistake.

## Conflict of interest statement

The author declares that the research was conducted in the absence of any commercial or financial relationships that could be construed as a potential conflict of interest.

